# Integrated prodrug micelles with two-photon bioimaging and pH-triggered drug delivery for cancer theranostics

**DOI:** 10.1093/rb/rbz035

**Published:** 2019-11-04

**Authors:** Hong Xu, Boxuan Ma, Jizhou Jiang, Sutong Xiao, Rongrong Peng, Weihua Zhuang, Gaocan Li, Yunbing Wang

**Affiliations:** 1 National Engineering Research Center for Biomaterials, Sichuan University, Chengdu 610064, China; 2 College of Polymer Science and Engineering, Sichuan University, Chengdu 610064, China

**Keywords:** aggregation-induced emission, two-photon, pH-sensitive, charge reversal, drug delivery system

## Abstract

Nanodrug carriers with fluorescence radiation are widely used in cancer diagnosis and therapy due to their real-time imaging, less side effect, better drug utilization as well as the good bioimaging ability. However, traditional nanocarriers still suffer from unexpectable drug leakage, unsatisfactory tumor-targeted drug delivery and shallow imaging depth, which limit their further application in cancer theranostics. In this study, an integrated nanoplatform is constructed by polymeric prodrug micelles with two-photon and aggregation-induced emission bioimaging, charge reversal and drug delivery triggered by acidic pH. The prodrug micelles can be self-assembled by the TP-PEI (DA/DOX)-PEG prodrug polymer, which consists of the two-photon fluorophore (TP), dimethylmaleic anhydride (DA) grafted polyethyleneimine (PEI) and polyethylene glycol (PEG). The PEG segment, DOX and DA are bridged to polymer by acid cleavable bonds, which provides the micelles a ‘stealth’ property and a satisfactory stability during blood circulation, while the outside PEG segment is abandoned along with the DA protection in the tumor acidic microenvironment, thus leading to charge reversal-mediated accelerated endocytosis and tumor-targeted drug delivery. The great antitumor efficacy and reduced side effect of these pH-sensitive prodrug micelles are confirmed by antitumor assays *in vitro* and *in vivo*. Meanwhile, these micelles exhibited great deep-tissue two-photon bioimaging ability up to 150 μm in depth. The great antitumor efficacy, reduced side effect and deep two-photon tissue imaging make the TP-PEI (DA/DOX)-PEG prodrug micelles would be an efficient strategy for theranostic nanoplatform in cancer treatment.

## Introduction

In recent years, chemotherapy is deemed to be one of the most important methods for cancer treatment [1, 2], while it also suffers from poor targeting, rapid clearance and severe side effects of antitumor drugs [3, 4]. To this perspective, drug-loaded nanocarriers, such as polymeric micelles, are utilized to achieve an efficient tumor inhibition with significantly reduced toxicity [5, 6]. With a particle size of about 100–200 nm, polymeric micelles exhibit extended circulation time in bloodstream and can accumulate at tumor tissue through the enhanced permeability and retention (EPR) effect [7–9], which would benefit an efficient drug delivery. Due to its enhanced therapeutic effect and obviously reduced adverse effect, drug-loaded polymeric micelles have shown great potential in tumor treatment.

However, several shortcomings still limit the bioapplication of drug-loaded micelles, such as undesirable drug leakage during body circulation, unsatisfactory endocytosis, nonspecific drug release and untraceable *in vivo* distribution [10–13]. As for the conventional micelles, the drugs are usually packaged *via* hydrophobic effect, which easily leads to the drug leakage during blood transportation [14]. Alternatively, the prodrug micelles provide a new strategy for an enhanced stability during the transportation in the bloodstream, where the drugs are conjugated to the micelles *via* covalent bonds [15–17]. Nevertheless, the problems that how to effectively release the drug to target sites and improve cellular internalization of drug-loaded micelles still remain. Furthermore, in order to realize good compatibility and stability in blood circulation, the micellar surface is always designed to be electronegative, which also suppresses the internalization of these micelles [18]. Thus, a tumor-specific drug release and a negative-to-positive charge conversion property are demanded when the micelles accumulate at tumor sites, which can be achieved by utilizing the specific tumor microenvironment [19, 20]. In view of the specific pH value of tumor tissue (6.2–6.8 in extracellular matrix) [21–23], the pH-sensitive structures, such as imine, orthoester and acetal, can be introduced into micellar construction to respond to tumor tissue microenvironment and result in the efficient drug release [15, 24–28]. Moreover, micelles with charge conversion ability would further enhance micellar endocytosis and improve antitumor effect [29, 30].

Besides the programmed pH-sensitive charge conversion and drug release, developing ‘visible’ nanocarriers is imperative for cancer diagnosis and evaluating the biodistribution of nanocarriers. Recently, the development of organic fluorescent probes for cancer diagnosis has become one of the hotspots of research [31, 32]. However, although the conventional organic fluorescent probes exhibit strong fluorescence intensity in dilute solution, with the increase of concentration and the formation of aggregates, the fluorescence emission is significantly weakened or even quenched, which is defined as the aggregation-caused quenching effect [33]. Fortunately, a plenty of fluorescent molecules such as hexaphenylsilole and tetraphenylethylene, which emits strong fluorescence in aggregated state, having been firstly reported by Tang and his co-workers in 2001, defined as the aggregation-induced emission (AIE) effect [34, 35]. This unique fluorescent property makes these AIE fluorescent probes potential candidates for tumor diagnosis. Moreover, traditional organic fluorescent probes are mostly single-photon excited, which is limited by ungratified penetration depth and the interference of autofluorescence [36–38]. Therefore, two-photon imaging system with strong penetration ability and high resolution in biological tissue has been developed with great bioimaging ability [39], which can be a great candidate for tumor diagnosis [40–42].

**Figure 1:**
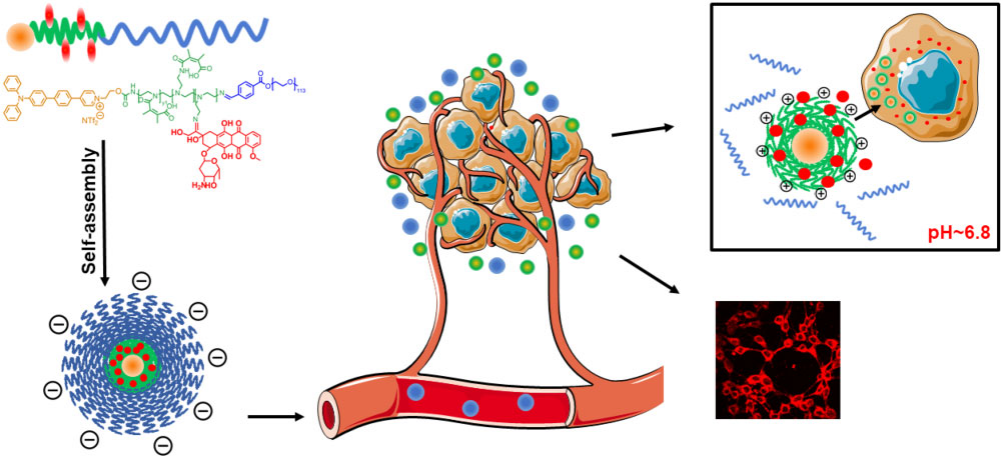
Illustration of TP-PEI (DA/DOX)-PEG prodrug micelles with charge reversal and drug delivery under acidic pH, as well as the two-photon excited AIE bioimaging

In this study, a comprehensive nanoplatform with charge reversal and drug release triggered by acidic pH, as well as two-photon AIE imaging capability has been constructed based on the two-photon fluorophore (TP) and doxorubicin (DOX) labeled prodrug copolymer TP-PEI (DA/DOX)-PEG, aiming for deep-tissue fluorescence bioimaging and effective tumor elimination (Fig. 1). DOX is conjugated with polymer by pH-sensitive imine bond, which ensures the micelles a good stability in the blood circulation with minimum drug leakage. However, after reaching the tumor tissue through the EPR effect, the prodrug micelles can be interrupted by the acidic microenvironment and the pH-sensitive bonds would be broken. The outmost polyethylene glycol (PEG) segment can be dropped as well as the grafted dimethylmaleic anhydride (DA) on polyethyleneimine (PEI) segment, causing the exposure of the amino groups on PEI. As a result, the micellar surface charge converts from negative to positive, which ultimately promotes the endocytosis of the prodrug micelles. Meanwhile, the conjugated DOX is started to be gradually released, which indicates the target-site drug release and an accurate tumor inhibition. Moreover, the micelles labeled by the two-photon AIE fluorophore developed in our earlier work can efficiently achieve the two-photon bioimaging with a strong fluorescence under a two-photon excitation [43]. The tumor-targeted charge conversion, accelerated drug release, as well as powerful two-photon fluorescence bioimaging demonstrate that these prodrug micelles can be a potential strategy for tumor theranostics.

## Material and methods

### Preparation of TP-PEI (DA/DOX)-PEG prodrug micelles

TP-PEI (DA/DOX)-PEG prodrug (10 mg) was dissolved in THF (1 ml), then the solution was added dropwise into stirring phosphate buffer solution (PBS, 7 ml). The mixture was allowed to stir in the dark at room temperature for 4 h. Then the solution was dialyzed against PBS (MWCO = 3500) for 24 h and extruded using a 0.22-μm filter to obtain the micellar solution (1 mg/ml). Moreover, blank TP-PEI (DA)-PEG micelles were developed by using the similar method without the conjugation of DOX. Dynamic light scattering (DLS) and transmission electron microscope (TEM) were used to characterize the micellar size and morphology.

### pH-response and *in vitro* drug release behavior of prodrug micelles

To evaluate the stability and pH sensitivity of these micelles, DLS was utilized to measure the zeta potential and particle size at different time intervals under pH 7.4 and 6.8. Moreover, micelle solution was treated by 1 M HCl for 24 h and lyophilized to measure the drug loading content (DLC) and drug loading efficacy (DLE) of the micelles by using UV-vis at 480 nm, where the lyophilized prodrug micelles were dissolved in methanol and DMF solution (1/1, v/v). Subsequently, the drug release behavior of the micelles was further characterized at pH 6.8 and 7.4, respectively. Micellar solution was equally distributed into two dialysis bags (1 mg/ml, 2 ml/bag, MWCO = 3500), which were incubated in 20 ml PBS at different pH (7.4 or 6.8) with constant shake in the dark at 37°C. Within the selected intervals, 2-ml sample was extracted and UV-vis at 480 nm was used to calculate the amount of released DOX.

### AIE behavior of prodrug micelles

The AIE property of prodrug micelles was investigated using fluorescence spectra, which was excited at 410 nm in solutions with various ratio of THF and water. The micelles remained at the same concentration in different ratio of solutions. In addition, the critical micelle concentration (CMC) of prodrug micelles was also evaluated by characterizing the AIE fluorescence under different micellar concentration (10^−6^, 5 × 10^−5^, 10^−5^, 5 × 10^−4^, 10^−4^, 5 × 10^−3^, 10^−3^, 10^−2^, 10^−1 ^mg/ml).

### *In vitro* tumor inhibition

The *in vitro* antitumor effect was characterized by MTT assays. 4T1 cells were seeded on 96-well plates (at a density of 5000 per well) and were incubated for 24 h with 200 μl medium. Afterwards, fresh medium (200 μl) at pH 6.8 or 7.4, which contained free DOX·HCl and prodrug micelles with DOX concentrations ranging from 0.1 to 10 μg/ml, was added to replace the original culture medium. After being incubated for another 48 h, 20 μl MTT solutions were added for another 4-h incubation of cells. Then 200 μl DMSO was used to substitute the medium was and the *in vitro* tumor inhibition ability were investigated by measuring the absorbance at 490 nm.

### *In vitro* cellular uptake and bioimaging

The cellular uptake would be promoted *via* the pH-sensitive charge reversal of prodrug micelles, which was evaluated by confocal laser scanning microscopy (CLSM). In brief, 4T1 cells were seeded on glass dishes and incubated for 24 h. And the prodrug micelles were added under pH 7.4 or 6.8 (40 μM TP), respectively. After an incubation for 2, 4 and 6 h, the cells were washed with PBS and observed with two-photon CLSM excited at 800 nm.

### *Ex vivo* fluorescence imaging

4T1 cells with a density of 10^6^ were injected on the right back of BALB/c mice to set up the subcutaneous tumor models. The solution of TP-PEI (DA)-PEG micelles without DOX conjugated (1 mM TP) was injected intravenously to mice *via* the caudal vein. The mice were sacrificed after 6, 12, 24 and 48 h, anatomized and the major organs (heart, liver, spleen, lung and kidney) were excised as well as tumors and observed using a Maestro *ex vivo* optical imaging system. In addition, the excised hepatic and nephric tissues were dyed at room temperature with Hoechst 33342 for 30 min after intravenous injection of TP-PEI (DA)-PEG micelles for 12 h. Collection of two-photon fluorescent images and Hoechst 33342 toward the nephric and hepatic tissues were obtained by utilizing CLSM excited at 405 and two-photon 800 nm.

### *In vivo* tumor inhibition

The subcutaneous tumor-bearing mice were randomly divided into three groups (six mice per group) when the volume of tumor approximately reached 100 mm^3^ (tumor volume *V* = 1/2 × *L* × *W*^2^. *L* and *W* stand for the length and width of tumor). Normal saline, free DOX·HCl and TP-PEI (DA/DOX)-PEG prodrug micelles were injected every 4 days with a total of four times (5 mg DOX/kg body weight), and the volume of tumor as well as the weight of body were measured every 2 days.

At the 21st day, all the mice were sacrificed. The major organs and tumors were excised and washed by PBS before being fixed with 4% formaldehyde. Afterwards, histopathological analysis was accomplished in virtue of slicing staining which dyed by hematoxylin and eosin (H&E).

### Immunohistochemical analysis of CD31 and TUNEL

The tumor sections were incubated against CD31 overnight at 4°C after rehydrated and deparaffined. And level two antibodies were used at 1:200 for 30 min before the addition of an egg protein reagent labeled with horseradish peroxidase.

For the TUNEL assay, tumor rehydration slices were incubated with protease K for 25 min at 37°C and rinsed with PBS for three times. Situ cell death detection kit-POD (Roche Group, Switzerland) was used for the TUNEL assay to evaluate cell apoptosis. Optical microscopy was used to observe the TUNEL staining, and the apoptosis index was calculated as the ratio of apoptotic cells number to the total number of tumor cells.

## Results and discussion

### Synthesis of TP-PEI (DA/DOX)-PEG prodrug polymer

**Figure 2:**
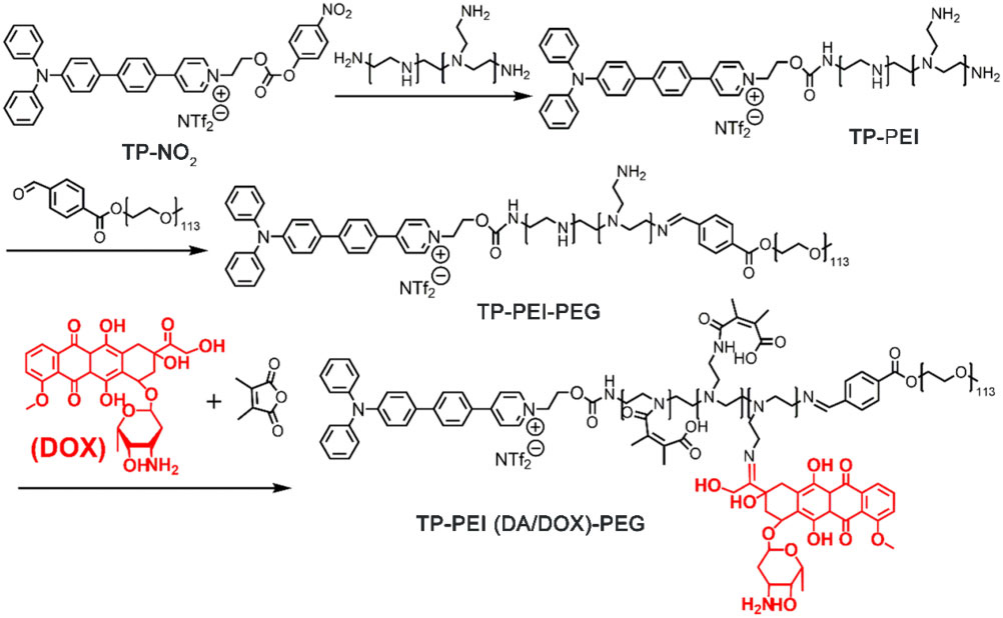
Synthesis route of TP-PEI(DA/DOX)-PEG prodrug polymer

**Figure 3:**
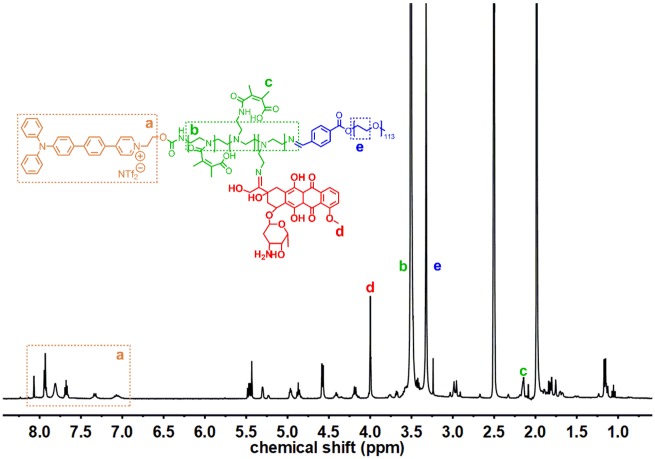
^1^H NMR Spectrum of TP-PEI (DA/DOX)-PEG in DMSO-*d_6_*

TP-PEI (DA/DOX)-PEG prodrug polymer was synthesized *via* several steps shown in Fig. 2. ^1^H NMR spectrum of TP-PEI was shown in Supplementary Fig. S1. Characteristic peaks belonging to TP (a, δ 7.0–8.0 ppm) and PEI (b, δ 3.4 ppm) were clearly observed, suggesting that TP was successfully bridged to the PEI and the molar ratio of TP and PEI was about 1:1 on the basis of the integral area ratio of peak a and peak b. The chemical structure of TP-PEI-PEG was also characterized by ^1^H NMR. The characteristic peak of PEG (c, δ 3.25 ppm) was discovered in Supplementary Fig. S2, where the integration ratio of peak b and c exhibited a ratio of approximate 1:1 between PEG and the TP-PEI. Furthermore, the great conjugation of DOX and DA was proven in Fig. 3. As shown in the ^1^H NMR spectrum in Fig. 3, the characteristic peak of DA (c, δ 2.18 ppm) and DOX (d, δ 4.00 ppm) could be easily discovered, suggesting the TP-PEI (DA/DOX)-PEG prodrug polymer was synthesized as expected.

### Preparation of TP-PEI (DA/DOX)-PEG prodrug micelles

**Figure 4:**
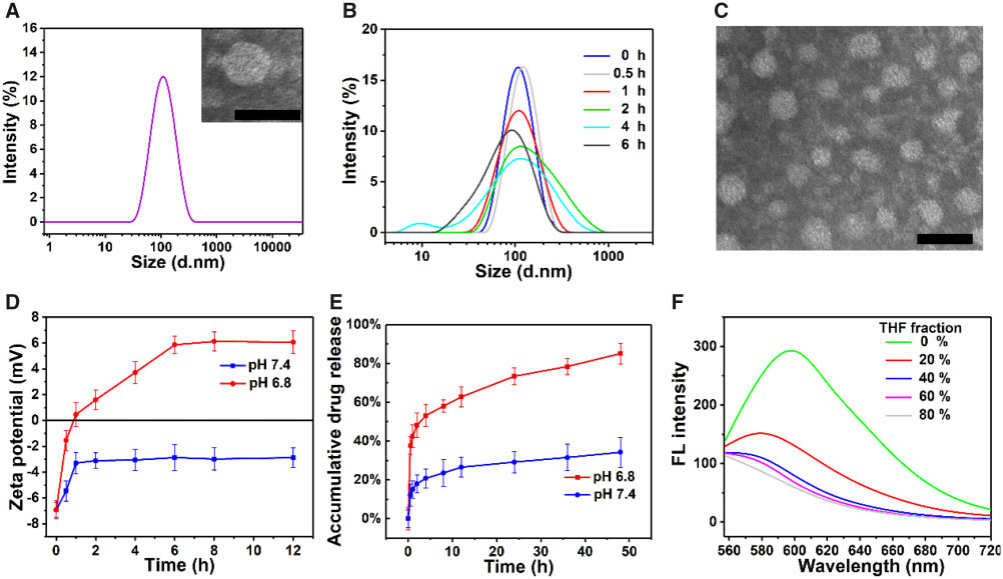
(**A**) Particle size of TP-PEI (DA/DOX)-PEG prodrug micelles measured by DLS and TEM image at pH 7.4. (**B**) Micellar size variation under acidic condition over time demonstrated by DLS. (**C**) TEM image of prodrug micelles under pH 6.8 for 6 h. (**D**) Changes of zeta potential at various pH with the extension of time tested by DLS. (**E**) *In vitro* accumulative drug release of prodrug micelles at various pH in 48 h measured by UV-vis. (**F**) FL spectrum of prodrug micelles in solution with various ratio of THF and water. The scale bars were 100 nm

TP-PEI (DA/DOX)-PEG prodrug could self-assemble into the micelles with a core-shell structure in aqueous media. The particle size of TP-PEI (DA/DOX)-PEG prodrug micelles was 98.0 nm and the size distribution (PDI) was 0.192 measured by DLS (Fig. 4A). Besides, the particle size of TP-PEI (DA)-PEG micelles without DOX conjugated was 101.0 nm with a PDI of 0.246 (Supplementary Fig. S3). In addition, the micellar morphology was detected by TEM, and a relatively uniform spherical morphology was observed (Fig. 4A), which was smaller than the DLS result due to the micellar shell shrinkage in sample preparation process. Furthermore, the CMC of prodrug micelles was characterized as 6.75 μg/ml (Supplementary Fig. S4), which indicated the micellar structure would be stable even being diluted by blood stream.

### pH-responsive behaviors

Due to the EPR effect, the TP-PEI (DA/DOX)-PEG prodrug micelles were expected to accumulate in tumor tissue. Afterwards, the acidic tumor microenvironment would trigger the charge reversal and accelerated drug delivery of these prodrug micelles. The TP-PEI (DA/DOX)-PEG prodrug micelles sowed a lasting stability for >96 h at physiological pH 7.4 (Supplementary Fig. S5). On the contrary, the size differentiation under acidic pH was observed and characterized by DLS. As expected, the size distribution of prodrug micelles changed from uniform to dispersive as shown in Fig. 4B. The reason for this size distribution transform was that the imine linkage on the micelles was broken under an acidic environment (pH ∼ 6.8) and PEG blocks gradually fell off as well as DOX and DA. Therefore, the TP-PEI would then re-assemble into a new micelle structure, which was also proven by the TEM results in Fig. 4C.

What’s more, while under acidic condition, the micellar zeta potential should change from negative to positive, which was due to the explosion of amino groups as the abandonment of PEG, DOX and DA. As shown in Fig. 4D, in the case of pH 7.4, the zeta potential was stable with a negative value, which ensured that the micelles maintained a ‘stealth’ state after entering the blood circulation. On the other side, when incubated in an environment of pH 6.8, the zeta potential rapidly raised from a negative value to over 0 mV within 1 h. Subsequently, the zeta potential continued growing up to +1.6, +3.7 and +5.9 mV at 2, 4 and 6 h, and eventually stabilized at about +6.1 mV after 6 h, respectively. As a result, the positive surface charge would make the micelles benefit to combine with the electronegative surface of cancer cells, which led to an enhanced endocytosis of prodrug micelles.

DOX was grafted to the TP-PEI-PEG copolymer *via* imine bond with a DLC of 12.9% and a DLE of 74.24%, and the breakage of imine linkage in micelles caused by an acidic pH would lead to an accelerated drug delivery at tumor site. To evaluate the drug release behavior of these prodrug micelles, the tumor acidic microenvironment was simulated in PBS at 37°C at pH 6.8. As shown in Fig. 4E, at pH 7.4, only 34% of the DOX was released after 48 h. In contrast, under pH 6.8, the release of DOX rapidly increased to >50% within 4 h and reached 85.1% after 48 h. Therefore, TP-PEI (DA/DOX)-PEG prodrug micelles were considered to possess good stability as well as great pH responsiveness, which would help to realize tumor-target drug delivery so as to enhance treatment efficacy.

### AIE behavior and two-photon cellular imaging

During the self-assembly of the prodrug micelles, the aggregation of TP would result in an AIE fluorescence. As shown in Fig. 4F, the prodrug polymer exhibited strong fluorescent emission in water. On the contrary, the fluorescence intensity rapidly decreased in the presence of THF. As increasing of THF fraction, the fluorescence intensity gradually weakened and the AIE fluorescence was totally quenched when the THF fraction was above 80%, which was due to the free molecular rotation of TP. The specific AIE fluorescent property of this prodrug micelle showed possibility to be a potential tool for *in vitro* and *in vivo* bioimaging.

**Figure 5:**
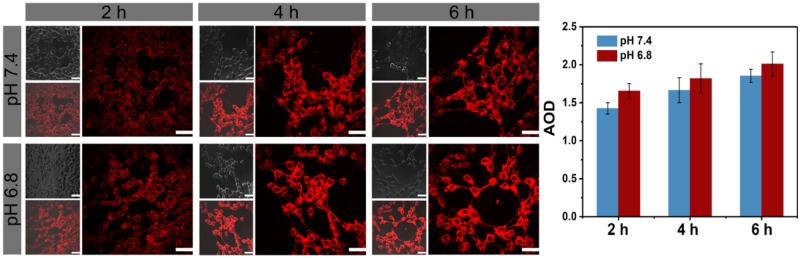
Two-photon imaging and average optical density (AOD) of 4T1 cells under CLSM excited at 800 nm, which were incubated with TP-PEI (DA/DOX)-PEG prodrug micelles for various time intervals and The scale bars were 100 μm

The two-photon cellular imaging ability of TP-PEI (DA/DOX)-PEG prodrug micelles was further observed by CLSM excited at 800 nm after co-incubation with 4T1 cells for 2, 4 and 6 h. As shown in Fig. 5, the two-photon fluorescence signal could be obviously observed after a co-incubation for 2 h. With the prolongation of time, more micelles were internalized, and the fluorescence intensity was getting stronger. Furthermore, cells cultured at pH 6.8 showed a stronger fluorescence in contrast to the cells at pH 7.4 in same time, which indicated that these pH-sensitive prodrug micelles with charge conversion under acid environment exhibited enhanced endocytosis to the tumor cells.

### *Ex vivo* fluorescence imaging

**Figure 6:**
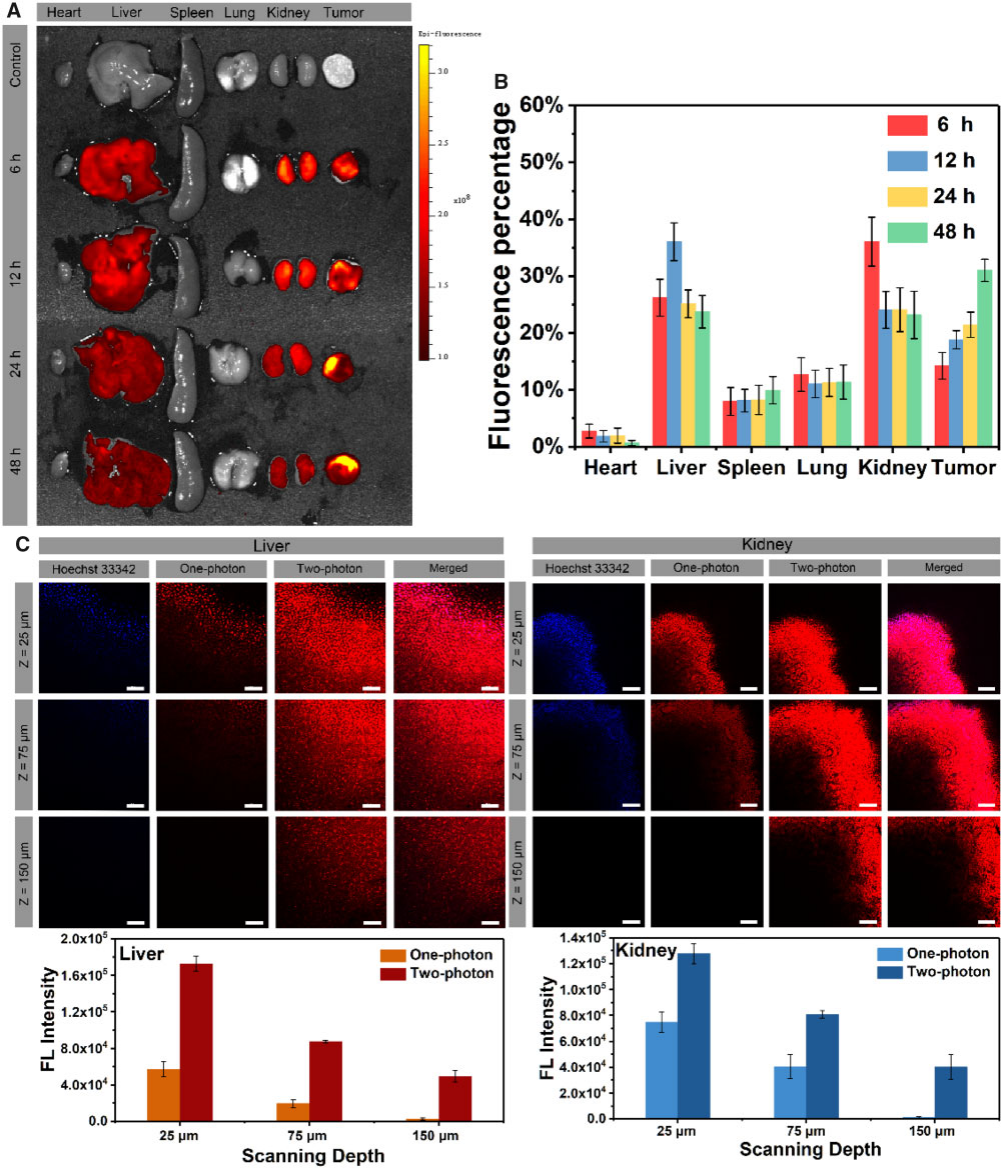
(**A**) Fluorescent images of *ex vivo* mean organs and tumors at different time postinjection. (**B**) Fluorescent intensity of TP-PEI (DA)-PEG micelles in major organs and tumors. (**C**) Confocal images and quantitative fluorescence intensity of kidney and liver tissues administrated with micelles for 12 h under two-photon excitation. The scale bars were 100 μm

The mice were sacrificed after injection of TP-PEI (DA)-PEG micelles at different time intervals, which served as an effective approach to determine the *in vivo* micellar distribution after injection. As shown in Fig. 6A, obvious fluorescent signal could be seen in livers, kidneys and tumors of mice as compared with the control group. The fluorescence intensity of tumors increased continuously within 48 h, which demonstrated that the EPR effect could be an effective means for accumulation of micelles at tumor site. In addition, degenerative fluorescence in the livers and kidneys indicated the micellar systemic metabolism. The variation of fluorescence intensity was further shown in Fig. 6B, which was in great agreement with the results in Fig. 6A. Thus, the prodrug micelles could be deemed as a strategy with great potential for accurate visibility of cancer treatment.

Furthermore, the bioimaging capability of TP-PEI (DA/DOX)-PEG prodrug micelles in deep-tissue bioimaging was characterized by two-photon CLSM. After 12 h of micellar injection, the nephric and hepatic tissues were excised and dyed with Hoechst 33342 immediately. In Fig. 6C, strong fluorescence was detected under 800 nm two-photon excitation in both nephric and hepatic tissues. As the scanning depth increased, the fluorescence of micelles and Hoechst 33342 under 405 nm one-photon excitation decreased dramatically. On the contrary, under two-photon excitation, fluorescent signals with a depth of up to 150 μm was still observed, indicating that TP-PEI (DA/DOX)-PEG micelles possessed a great potential for deep-tissue bioimaging upon two-photon excitation.

### *In vitro* tumor inhibition

**Figure 7:**
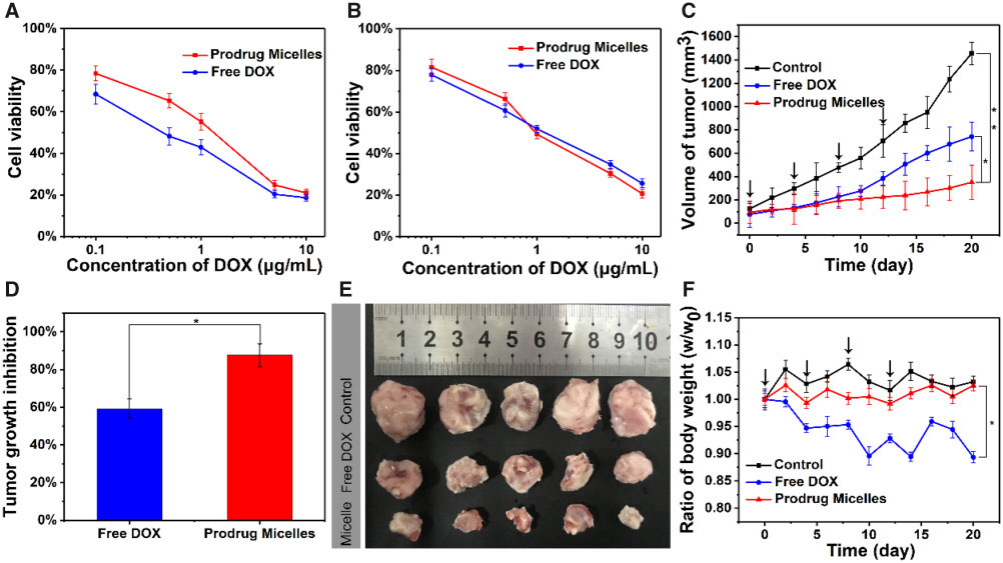
Cytotoxicity of 4T1 cells co-cultured with different concentrations of free DOX and TP-PEI (DA/DOX)-PEG prodrug micelles at pH 7.4 (**A**) and 6.8 (**B**) for 48 h. (**C**) The volume of tumors treated with saline, free DOX and TP-PEI (DA/DOX)-PEG prodrug micelles over 3 weeks (*P <* *0.05, **P <* *0.01). (**D**) The ratio of tumor growth inhibition treated with TP-PEI (DA/DOX)-PEG prodrug micelles and free DOX over 3 weeks (*P <* *0.05). (**E**) The photograph of tumors treated with saline, free DOX and TP-PEI (DA/DOX)-PEG prodrug micelles over 3 weeks. (**F**) Mice body weights ratio (real-time weight/initial weight), which were treated with saline, free DOX and TP-PEI (DA/DOX)-PEG prodrug micelles in 3 weeks (*P <* *0.05; black arrows represented the drug injection)

As the nanocarriers with pH-triggered drug release, TP-PEI (DA/DOX)-PEG prodrug micelles were supposed to possess an outstanding tumor inhibition ability, which was characterized by MTT assays. As shown in Fig. 7A, with the concentration of DOX increased from 0.1 to 10 μg/ml, the 4T1 cell viability reduced from 78% to 22% at pH 7.4 in 48 h, while free DOX showed better tumor cells inhibition ability due to the difference in the cellular uptake pathway. On the other side, the cells viability was decreased to 18% after the treatment with prodrug micelles for 48 h at pH 6.8 while the DOX concentration was 10 μg/ml (Fig. 7B), which showed comparative tumor cell elimination as compared with free DOX. This might be attributed to the acid-induced charge reversal-mediated enhancement cellular uptake of micelles.

### *In vivo* antitumor efficacy

In order to further investigate the *in vivo* tumor suppression ability of TP-PEI (DA/DOX)-PEG prodrug micelles, free DOX and TP-PEI (DA/DOX)-PEG prodrug micelles were used for a treatment of 4T1 breast cancer BALB/c mice after the inoculated volume of the tumor reached about 100 mm^3^, and saline was used as the control group. As shown in Fig. 7C, the control group did not show any tumor inhibition effect with a rapidly increased tumor volume. As compared with saline and free DOX, the prodrug micelles exhibited a more intensive ability to inhibit tumor growth. Moreover, the ratio of tumor growth inhibition treated with TP-PEI (DA/DOX)-PEG prodrug micelles was also significantly higher than that treated with free DOX (Fig. 7D). Moreover, the difference in the size of tumors treated with saline, free DOX and TP-PEI (DA/DOX)-PEG prodrug micelles could be clearly observed from Fig. 7E, which was in keep with the result in Fig. 7C. As a result, it could be demonstrated that the prodrug micelles possessed an excellent *in vivo* tumor inhibitory effect. Furthermore, to evaluate DOX-induced toxicity, body weight loss was monitored and the result was shown in Fig. 7F. After 21 days of treatment, a significant weight loss which is about 11% of body weight was recorded in the free DOX-treated mice. However, no obvious changes were observed in weight of mice injected with prodrug micelles as well as saline, indicating an ideal biocompatibility of TP-PEI (DA/DOX)-PEG prodrug micelles.

### Histological studies and immunohistochemical analysis

**Figure 8:**
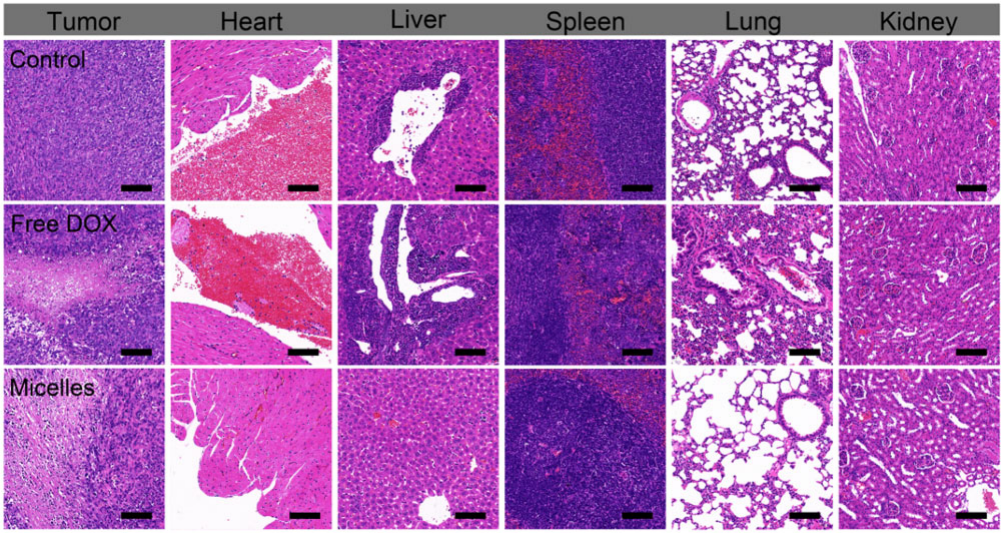
H&E sections of hearts, livers, spleens, lungs, kidneys and tumors after treated with saline, free DOX and TP-PEI (DA/DOX)-PEG prodrug micelles over 21 days. The scale bars were 100 μm

Histological studies were used to verify the splendid biocompatibility and great therapeutic efficacy of TP-PEI (DA/DOX)-PEG prodrug micelles. As shown in Fig. 8, infiltration of inflammatory cells and focal thrombosis could be found in section of heart, liver, spleen and lung of mice under H&E staining, which treated with free DOX, thus suggesting serious organ toxicity of DOX. On the contrary, few inflammation and toxicity were discovered in H&E section of main organs in prodrug micelles-treated mice due to their satisfactory biosecurity. Furthermore, significantly extensive apoptosis of tumor cells was observed in tumor treated with prodrug micelles, which confirmed the outstanding antitumor effect of these prodrug micelles.

**Figure 9:**
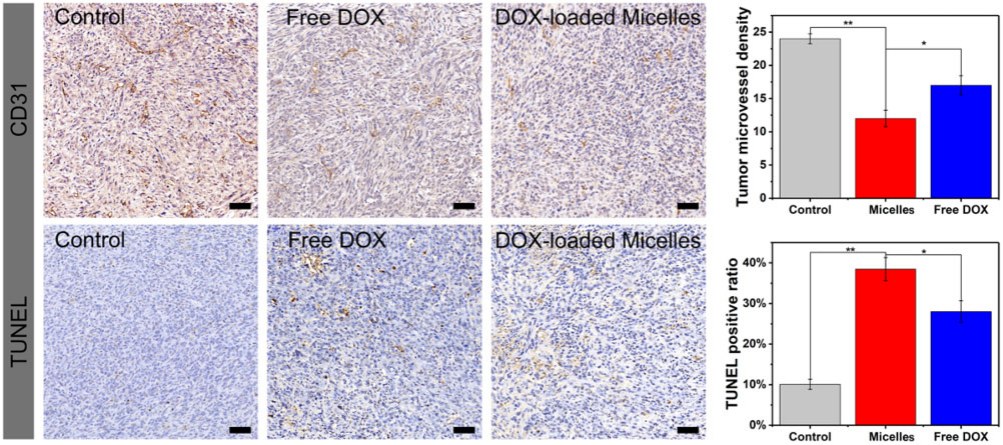
CD31 and TUNEL Immunohistochemical (IHC) staining of tumor tissues treated with saline (control), free DOX and TP-PEI (DA/DOX)-PEG prodrug micelles. The brown areas demonstrated CD31-positive and TUNEL-positive staining. The scale bars were 20 μm. The capillary number was counted for CD31 (**P <* *0.01, *P <* *0.05). The proportion of apoptotic cells was calculated as the apoptotic indices (**P <* *0.01, *P <* *0.05)

The antitumor ability of TP-PEI (DA/DOX)-PEG prodrug micelles was further characterized by immunohistochemical analysis (CD31 and TUNEL). Tumor metastasis and invasion was mainly caused by angiogenesis in the tumor tissue, which could be characterized by CD31 assay. Moreover, the tumor apoptosis could be investigated via TUNEL assay. As shown in Fig. 9, compared with the groups treated with free DOX and saline, fewest angiogenesis and most apoptotic cells were found in the micelle-treated group, respectively. On the basis of this, it could be speculated that the rapid accumulation, efficient endocytosis and rapid intracellular drug release of TP-PEI (DA/DOX)-PEG prodrug micelles could effectively inhibit tumor angiogenesis and cell apoptosis, leading to a satisfactory antitumor efficiency.

## Conclusion

In this work, the prodrug micelles with two-photon AIE fluorescent bioimaging, tumor acidic microenvironment triggered charge conversion and drug release have been designed for bioimaging and therapy. The TP-PEI (DA/DOX)-PEG prodrug micelles exhibit good stability and can accumulate effectively at the tumor site through the EPR effect. Furthermore, the acidic environment of the tumor tissue can trigger the charge conversion and accelerate the drug release so as to promote the endocytosis along with the enhanced tumor inhibition. Although the prodrug micelles show a great antitumor ability with minimal side effects *in vivo*, a deep-tissue bioimaging under two-photon excitation up to 150 μm has also been demonstrated, which show great potential for tumor fluorescent diagnosis. Overall, the development of the TP-PEI (DA/DOX)-PEG prodrug micelles provides a new prospective for cancer theranostic application.

## Funding 

This research was financially supported by the National Natural Science Foundation of China (Projects No. 21502129), the National 111 Project of Introducing Talents of Discipline to Universities (No. B16033), China Postdoctoral Science Foundation Funded Project (Nos. 2017M612956, 2018T110969) and the State Key Laboratory of Polymer Materials Engineering (No. sklpme2018-3-05). We would be grateful to the help of Mr. Chenghui Li (Analytical & Testing Center, Sichuan University) of taking laser scanning confocal images. 

*Conflict of interest statement*. None declared.

## Supplementary Material

rbz035_Supplementary_Data
