# Supplementary material for: Integrated prodrug micelles with two-photon bioimaging and pH-triggered drug delivery for cancer theranostics
Source: Regen Biomater. 2019 Nov 4;7(2):171–80. doi: 10.1093/rb/rbz035 (PMC7147369; doi:10.1093/rb/rbz035)
Supplement: rbz035_Supplementary_Data [file rbz035_supplementary_data.zip › rbz035-Suppl_Data/Supporting Information.docx]

**Support Information**

**Integrated Prodrug Micelles with Two-photon Bioimaging and pH-triggered Drug Delivery for Cancer Theranostics**

*Hong Xu, Boxuan Ma, Jizhou Jiang, Sutong Xiao, Rongrong Peng, Weihua Zhuang*, Gaocan Li* and Yunbing Wang*

*Hong Xu* and *Boxuan Ma* contributed equally in this work.

*Hong Xu, Boxuan Ma, Weihua Zhuang*, Gaocan Li* and Yunbing Wang*

National Engineering Research Center for Biomaterials, Sichuan University, Chengdu 610064, China

E-mail: weihua-zhuang@qq.com (Weihua Zhuang); gaocanli@scu.edu.cn (Gaocan Li)

*Jizhou Jiang, Sutong Xiao, Rongrong Peng*

College of Polymer Science and Engineering, Sichuan University, Chengdu 610064, China

**1 Materials**

Polyethylene imide (PEI), doxorubicin (DOX··HCl) and dimethylmaleic anhydride (DA) were obtained from Adamas Reagent, Ltd (Shanghai, China). MTT was purchased from Chengdu Best Reagent Co., LTD (Chengdu, China). Dimethyl formamide (DMF), tetrahydrofuran (THF) and all other reagents and solvents were purchased from Chengdu KeLong Chemical Reagent Company (Chengdu, China) and used without further purification. Polyethylene glycol (PEG_5000_-CHO) and two-photon fluorophore with nitrophenyl ester (TP-NO_2_) were synthesized according to our previous study^[40]^. The synthesis of the prodrug polymer TP-PEI (DA/DOX)-PEG was shown in Scheme 2.

**2 Synthesis of TP-PEI polymer**

Polyethylene imide (PEI, M_w_ = 1800 g/mol, 0.1125 g, 0.0625 mmol) was dissolved in THF with TEA (0.026 ml, 0.1875 mmol). Then TP-NO_2_ (50 mg, 0.0625 mmol) was dissolved in THF and added dropwise. The reaction system was stirred for 24 h at room temperature, then the solution was allowed to dialyze against deionized water (MCWO = 2000) for 24 h, and obtaining TP-PEI polymer *via* freeze-drying as a result.

**3 Synthesis of TP-PEI(DA/DOX)-PEG prodrug polymer**

TP-PEI (40 mg, 0.015 mmol) was dissolved in THF, and polyethylene glycol with benzene formaldehyde sealing end (PEG_5000_-CHO, 77mg, 0.015 mmol) was added into the solution. After stirred at 40 ^o^C for 48 h, the reaction solution was dialyzed (MWCO = 5000) for 24 h against deionized water, followed by lyophilization to get TP-PEI-PEG polymer.

For the DA and DOX conjugation, DOX·HCl was neutralized with TEA in DMF, thus preparing doxorubicin (DOX) firstly. Afterwards, TP-PEI-PEG copolymer (76 mg, 0.01 mmol) and dimethylmaleic anhydride (DA, 38 mg, 0.30 mmol) were dissolved in THF along with 38 mL of DOX solution (4 mg/mL) and stirred in the dark at room temperature for 24 h. Afterwards, the solution was dialyzed against deionized water (MWCO = 5000) in the dark for 24 h, followed by freeze-drying to obtain the TP-PEI (DA/DOX)-PEG prodrug polymer.

**4 Characterization**

The ^1^H NMR spectra were obtained by a spectrometer operating at 400 MHz (Bruker AMX-400). Dynamic light Scattering (DLS) on a Malvern Zetasizer Nano ZS was used to record the particle size and zeta potential of the prodrug micelles. The sample was stained with 2% phosphotungstic acid before measuring and observed by a Hitachi transmission electron microscope (TEM) with the accelerating voltage of 80 KV.

**5 Cell culture**

The murine breast cancer (4T1) cells were cultured in 5% CO_2_ atmosphere at 37 ^o^C using RPMI 1640 medium supplemented with 1% (v/v) penicillin-streptomycin and 10% (v/v) fetal bovine serum (FBS), respectively.

**6 Animal experiments**

All animal experiments were approved by the Sichuan Provincial Committee for Experimental Animal Management and carried out in accordance with the guidelines of the institutional and the NIH on the study of animal care and use. BALB/c mice (female, 20-25 g) were provided by West China Experimental Animal Center of Sichuan University (China) and housed at a 50-60 %relative humidity, 20-22 ^o^C temperature and 12 h light-dark cycles with deionized water ad libitum and commercial mice pellet diet.

**7 Statistical analysis**

All experiments were conducted at least three times and expressed as means ±SD. The Student’s t test was used to analyze the statistical significance between each group, where *p* values of < 0.05 indicated statistical significance.

**

**

**Figure S1.** ^1^H NMR spectrum of TP-PEI in DMSO-*d_6_*.

**

**

**Figure S2.** ^1^H NMR spectrum of TP-PEI-PEG in DMSO-*d_6_*.





**Figure S3**. Particle size of TP-PEI (DA)-PEG micelles without DOX conjugated measured by DLS at pH 7.4.





**Figure S4**. CMC determination for TP-PEI (DA/DOX)-PEG prodrug micelles.





**Figure S5**. Stability of TP-PEI (DA/DOX)-PEG prodrug micelles in PBS.
